# Bioethics in an oncological surgery unit during the COVID-19 pandemic: the Verona experience

**DOI:** 10.1007/s13304-022-01279-5

**Published:** 2022-03-17

**Authors:** Massimiliano Tuveri, Claudio Bassi, Alessandro Esposito, Luca Casetti, Luca Landoni, Giuseppe Malleo, Giovanni Marchegiani, Salvatore Paiella, Martina Fontana, Matteo De Pastena, Pea Antonio, Giampaolo Perri, Alberto Balduzzi, Enrico Polati, Gabriele Montemezzi, Katia Donadello, Beatrice Milan, Salvatore Simari, Domenico De Leo, Beatrice Personi, Veronica Marinelli, Kathrin Ohnsorge, Veronica Adda, Roberto Salvia, The Verona Pancreas Institute Bioethics Multidisciplinary Group

**Affiliations:** 1 General and Pancreatic Surgery Unit, Verona Hospital Trust, University of Verona, Verona, Italy; 2 Intensive Care Unit, Verona Hospital Trust, University of Verona, Verona, Italy; 3 Department of Diagnostics and Public Health, Section of Forensic Medicine, University of Verona, Verona, Italy

**Keywords:** Covid 19 pandemic, Pancreatic surgery, Pancreatic tumors, Surgical triage, Ethics

## Abstract

The spread of COVID-19 has overwhelmed medical facilities across the globe, with patients filling beds in both regular wards and in intensive care units. The repurposing of hospital facilities has resulted in a dramatic decrease in the capacity of hospitals—in terms of available beds, surgical facilities, and medical and nursing staff— to care for oncology patients. The Italian National Board of Bioethics provided precise and homogeneous guidelines for the allocation of the scarce resources available. In our experience, strictly following these general guidelines and not considering the clinical vocation of each single health care center did not allow us to resume usual activities but generated further confusion in resource allocation. To face the scarcity of available resources and guarantee our patients fair access to the health care system we created a surgical triage with four fundamental steps. We took into consideration “ well defined and widely accepted clinical prognostic factors ” as stated by the Italian Society of Anesthesia and Resuscitation. We were able to draw up a list of patients giving priority to those who theoretically should have a greater chance of overcoming their critical situation. The age criterion has also been used in the overall evaluation of different cure options in each case, but it has never been considered on its own or outside the other clinical parameters. Although not considered acceptable by many we had to forcefully adopt the criterion of comparison between patients to give priority to those most in need of immediate care.

## Introduction

All over the world the spread and need of care of COVID-19 cases have overwhelmed the hospital facilities with patients filling beds in both regular wards and in intensive care units (ICUs) [1]; almost all structural, human, instrumental and economic resources have been allocated for the pandemic [2]. These reconversions and repurposing of hospital facilities has resulted in a dramatic decrease in the capacity of hospitals to care for oncology patients [3, 4].

Although the dispositions of our local Veneto Regional Authorities in March 2020 (protocol number 120472) [5], renewed in November 2020 (protocol number 474775) [6], ordered the temporary suspension of elective surgical activity, the same dispositions ensured the full operation of both medical and surgical oncological activities. However, the pandemic began placing extraordinary demands on medical personnel that would have been involved in these activities in ordinary times, such as anesthetists, diverting them to the management of COVID-19, thereby reducing the availability of operative sessions/slots [3, 7]. The immediate and net effects produced by the lack of available medical staff have tripled our surgical waiting list and doubled the outpatient waiting list for invasive procedures requiring anesthesia support.

Due to this unexpected situation, each surgical department, depending on its clinical vocation, has autonomously tried to determine different criteria and actions to address the increasing need for care and to allocate the resources available in the best possible and most rational way [8].

Certain principles for a simple basic surgical triage have suggested delaying pancreatic pathologies with a low degree of biological aggressiveness compared to duct carcinoma such as, for example, cystic or neuroendocrine tumors.

Actually, resource allocation principles in case of scarce medical resource are a complex, persistent ethical challenge, and have been already extensively described in literature [9, 10]. Starting from simple allocation principles, Persad and colleagues [9] described an original allocation system which prioritizes younger people who have not lived a complete life, and incorporates “prognosis”, “save the most lives”, “lottery”, and “instrumental value” principles. The same authors recently proposed six specific recommendations for allocating medical resources in the COVID-19 pandemic: 1, maximize benefits; 2, prioritize health workers; 3, do not allocate on a first come, first served basis; 4, be responsive to evidence; 5, recognize research participation; and 6, apply the same principles to all COVID-19 and non-COVID-19 patients [10].

To provide more precise and homogeneous directives, the Italian National Board of Bioethics (INBB), stated that “on the basis of the constitutional principles concerning the right of health care, equality principles, obligation of solidarity, and the universal and egalitarian criteria on which our health care system is founded, the INBB believes that the allocation of the resources must respect the principles of justice, equity and solidarity.” [11, 12]

In this context, it is essential for the clinician to interface with the bioethicist. In this paper, we try to report the path we have taken to give an answer as ethical as possible in the choice of priorities.

## Clinical criteria

The clinical criterion was recognized by the INBB as the most important for the allocation of resources, believing that other criteria, such as age, sex, social position, ethnicity, social role, physical impairment, lifestyle, and comparison of patients, were not justifiable from an ethical point of view. Based on this criterion, each individual patient had to be considered individually with the same attention given to assessing his or her needs, the expected benefit, and the appropriateness and proportionality of the potential risks associated with the treatment.

In our experience, strictly following these general guidelines and not considering the clinical vocation of each single health care center did not allow us to resume usual activities but generated further confusion in resource allocation. For example, delaying pancreatic pathologies with a low degree of biological aggressiveness compared to ductal cancer, as we stressed before, can be a questionable choice on the level of both “expected benefit” and “proportionality” of the treatment related risks.

Our experience has been the same as other medical associations—the Italian Society of Anesthesia and Resuscitation (SIAARTI) and the Forensic Medicine Board (SIMLA)—that in their documents have clearly suggested the need to interpret and adapt these guidelines to every specific center [12].

Often, resource scarcity can be alleviated by improved efficiency or expanded investment.

As stressed by the COVIDSurg Collaborative community [4] the surgery system worldwide was fragile to lockdowns with one in seven patients not undergoing planned surgery and longer preoperative delays, in particular for both esophageal and pancreatic cancer. Therefore, during social restriction for the next future it will be necessary to strength and protect surgical elective staff and services.

However, if these solutions cannot solve the problem if not in medium–long time, “rationing” is an option that demands making serious choices that are never easy. Facing the resource dilemma, both SIAARTI and SIMLA edited a document that states the need to give priority of access to ICUs to those patients who have a good prognostic outcome based on scientific knowledge. [12]

The evaluation of each patient is meant to stratify the possibility of overcoming the critical phase of an ailment through an intensive care approach and “will be based on the overall evaluation of each patient considering the number and type of comorbidities; previous functional status and frailty relevant to the response to the required treatment; severity of the current clinical status; the presumed impact of intensive care treatments, also in consideration of the patient's age; and the will of the sick person regarding intensive care treatments, which should be investigated as soon as possible in the initial phase of triage.”

In their documents, SIAARTI and SIMLA pointed out that both the chronologic criterion (order of arrival or date of enrollment on the waiting list) and the casual sorting had to be banned from the triage criteria, since they are not ethically acceptable, and they do not guarantee that limited resources are given to those who actually need them most as determined on a clinical basis.

To avoid misunderstanding, the document states that the patient’s age “has to be taken into account as a part of the whole patient’s assessment with no strict cutoff values”. Only when other parameters overlap may the patient’s age be considered as a discriminating value, as with increasing age, the probability of responding to intensive care decreases.

## Specificity and surgical triage at the Verona Pancreas Institute

Since the 1970s, the Pancreas Institute of the Verona University Hospital Trust has been devoted to the research, diagnosis, and care of patients with pancreatic diseases. Over time, the main interest of the Institute has been focusing on cancer, which currently represents 95% of our surgical activity [13]. While surgical procedures on noncancer patients were suspended indefinitely, these suspensions did not increase our surgical capacities for cancer patients. We have documented the increase in the number of patients on our surgical waiting list and helplessly witnessed the definitive loss of the opportunity to undergo surgery for many of our patients. It is not difficult to understand what this has entailed and continues to mean in terms of psychophysical stress for patients, relatives, and health and administrative personnel, as well as the relational repercussions for all involved [14].

The need to face the scarcity of available resources and guarantee our patients fair access to the health care system has given us the chance to optimize quality and quantity of the cure to achieve the best possible benefit. Aware of the need to apply personal judgment and knowing that scientifically supported guidelines were still not very clear, we were exploring something new and unfamiliar.

Our approach (“Four Steps”) involved four fundamental steps. In the first, a score was assigned to each patient with the aim to compare homogeneous patients [Table 1]. After medical examination, to plan the resources necessary to better treat the patient without neglecting any aspect of his or her management, we verified if all resources were available as stated by SIAARTI in its document [12], by considering “well defined and widely accepted clinical prognostic factors”.

**Table 1:** The “Four Steps” of surgical triage

Steps	Scoring	
	Yes	No
1 = Prognostic factors		
Physical and psychological fitness for surgery	1	0
Surgical success probability	1	0
Expected survival	1	0
Quality of life	1	0
If similar step 1 score:		
2 = Treatment urgency		
Surgery vs other treatments	1	0
Patient’s perspective and will	1	0
If similar step 2 score:		
3 = Therapeutic resources needed		
Prioritize who need less to optimize OR time	1	0
If similar step 3 score:		
4 = First come, first served		
Prioritize the order of presentation	1	0

The parameters we considered for the score were physical and psychological fitness for surgery, success of the procedure, expected survival and quality of life. We have tried in this way to evaluate the actual patient’s advantages by subtracting the disadvantages of such a complex procedure.

As already stressed in the previous “clinical criteria” paragraph, in the second step, we considered, among patients with equal or similar score, the urgency of treatment comparing the different potential aggressiveness of the underlined disease (i.e., pancreatic ductal adenocarcinomas vs. neuroendocrine or cystic tumors) and the possibility of placing the patient into “alternative” options such as chemo and or radiotherapy or ablative procedures, potentially able to act as a time bridge while waiting for resources to become available again.

Along with these clinical criteria, we also considered the patients’ will once he or she was given complete information about the disease course. For these reasons, the patient’s wills were investigated frequently, and new evaluations were made at every change in his or her clinical status. In the third step, we then compared the commitment of therapeutic resources required between among homogeneous patients to prioritize who needed the fewest resources so that we could operate on as many patients as possible, trying to concentrate the procedures with less time consumption in the same operating session or perform tumor ablations in sufficiently protected radiological environments instead of in the operating theater.

The fourth step determined an order of treatment among patients with the same or similar score after the first three steps, according to a “first come, first served” policy.

## Discussion

Rationing in medicine is an everyday “real life” necessity. Rationing is not always ethical [12, 15] but it can rightly become so such in a specific context where it is not possible to make the system more efficient or invest more resources.

The distribution of resources arises at two different and complementary levels: first, the organization of public assistance with general rules inspiring its management (macro-allocation); and second, the choice of everyday practice criteria for individual healthcare professionals deciding on the use of the means available and facing of an excessive request (micro-allocation). It is neither our purpose nor our competence to discuss macro-allocation here: we certainly wish that the amount of resource devotes to health, in relation to other budget chapters, will be greater soon and this both directly and indirectly by acting on living conditions, disease prevention and increasing the economic commitment for scientific research: the pandemic, at least this, should have taught us!

In our daily life, the micro-allocation of resources then becomes essential and requires difficult choices, which are not always unanimously acceptable into the dilemma between ethics and economics.

The choice of some clinical parameters, the comparison of patients, and the consequent human and social repercussions are often necessary, considering the specific vocation of each operating unit.

According to these criteria, we were able to draw up a list of patients giving priority to those who theoretically should have a greater chance of overcoming their critical situations. Although not considered acceptable by many [1, 12], we had to forcefully adopt the criterion of comparison among patients to give priority to those most in need of immediate care. In this scenario, the adoption of the comparison criterion was meant to achieve the best outcome possible and not to discriminate between cancer patients.

We also examined the quality of life of each individual patient very carefully based on a previous study [14] and after probing the patient about his or her postoperative expectations. Physicians often rate the patient’s quality of life much lower than the patient does. For this reason, the patient was subjected to several interviews with a psychologist so that we understood and did not neglect his or her real expectations.

The age criterion has also been used in the overall evaluation of different cure options in each case, but it has never been considered on its own or outside the other clinical parameters.

In pancreatic surgery, in similar clinical settings, younger age should favor a better and more satisfactory surgical outcome; however, it is also a condition that favors the development of severe postoperative complications, such as pancreatic fistulas (the “young” pancreas tends to be soft and delicate, while the “old” pancreas is more often hard, fibrotic and resistant to inevitable surgical manipulations), giving rise to a long recovery time and therefore a long hospitalization period [16, 17]. This development would, thus, increase the wait time for other patients on the waiting lists.

For these reasons, age was always evaluated together with other factors and especially the possibility of a favorable outcome, forcing us to discriminate between clinically similar patients to obtain the best possible effect from the treatment. This has been done in accordance with strict suitability and in proportion to the cure criteria, giving great importance to patients with a greater chance of overcoming the critical phase of illness and having an “acceptable lifetime expectation” [12].

We have always taken into consideration possible alternative strategies for those patients to whom we could not guarantee the due treatment at the due moment. We referred patients to chemo/radio/ablative treatments when these were viewed as possible alternatives [18]. Furthermore, whenever possible and feasible for the patient, we shifted them to other hospitals that could secure the same standard of care using all their available resources.

Patient priority was always decided during multidisciplinary weekly meetings: the Multidisciplinary Oncologic Pancreas Group; the pancreas round [19]; the Multidisciplinary Diagnostic Radiotherapy Gastroenterology Group; the Multidisciplinary Neuroendocrine Tumor Group; the Rare Tumor Group; and the Multidisciplinary Bioethics Group. Priority criteria have been flexible and reconsidered on a weekly basis, according to new possible resources available [3, 7]. An ongoing review of the waiting list during weekly multidisciplinary meetings has favored this procedure in the transparent attempt to offer the few resources (operating room and ward beds) to those who could truly benefit from them. We have not only considered patients already on the waiting list, as the rules for triage require, but we included new patients who were fully informed whenever surgical treatment was needed.

We are fully aware that every choice, even though taken on the basis of both scientific knowledge and ethical criteria based on justice, solidarity and transparency, can inevitably be considered some kind of discrimination and inequity in this scenario [20].

As long as we are going to be in a pandemic condition in our setting, it is and it will be very difficult, if not impossible, to combine the best outcome for the patient with the scarce resources available. Furthermore, the awareness of having dramatically passed from a substantially limitless functional autonomy to a vulnerable reality from a physical, moral, social, and political point of view weighs on our decision-making skills. We are suffering increasingly from a feeling of frustration and abandonment, which makes us less capable of facing our daily routines that now include finding good solutions to problems without having the resources to do so [3, 7]. This decision-making ability is also impaired by the spasmodic and continuous search for medico-legal protection due to the fear of future litigation because many planned treatments were not performed. Many patients did not receive the right treatment at the right time, with inevitable disease progression becoming inoperable or leading to an important prognostic worsening. Additionally, the CNB in many of the documents edited lately has highlighted the worrying increase of litigation, with the hope that a new set of laws will be made to protect health care providers from being sued in this pandemic framework. Nevertheless, we think that many of the potential changes in our legal system will have to do only with the management of COVID-19 patients, that is, treatment and medication, which are not yet approved universally by the scientific community [11]. In turn, recent scientific studies have stressed the importance of “collateral damage” due to delayed oncologic treatments during the pandemic, citing patients with a substantial and documented prognostic worsening because of only a 4-week delay in surgical treatment [21–26]. If we consider the “loss of chances” in the category of compensable damage in civil health liability [27], the concern of possible legal disputes is not part of the risk but risks soliciting "defensive medicine" that we would like to always oppose.

We were eager to find medical team members with competence in bioethics that would be integrated into our staff and willing to obtain a comprehensive understanding and agreement among the persons taking part in this new enterprise regarding the ethical dilemmas involved in this emergency and possible future repercussions. Anxious to find skills in bioethics, serenity and homogeneity of judgment in among staff members, we considered it necessary to create a working group including surgeons, anesthetists, nurses, psycho-oncologists, coroners, and bioethics experts called the Multidisciplinary Bioethics Group (BEG) and to draft a guiding document.

The main aim of the group was not to find answers to the many ethical problems and questions we face daily but to possibly plan an educational pathway to answer heath professionals’ questions and to provide them with all the tools they needed to make fair and equitable decisions regarding the patients’ best clinical outcomes.

The “BEG” multidisciplinary group was fundamental in outlining the path of the “four steps” approach where the various competences confronted each other to find the parameters of greater balance and common sense possible.

This has represented and still represents an element of great mitigation of our decision-making inner troubles of conscience, restoring us greater serenity and, we believe, complete professional capacity.

The Anglo-Saxon root of the word beg means to “to implore”. It is not an accident that this is our group’s acronym since the topic we are dealing with intrinsically begs for mercy as we attempt the evident human impossibility of achieving perfection of judgment and indisputable ethical behavior.

## References

[1] Italian Ministry of Health. www.salute.gov.it/nuovocoronavirus.

[2] Remuzzi A, Remuzzi G (2020). COVID-19 and Italy: what next?. Lancet.

[3] Marchegiani G, Perri G, Bianchi B, Esposito A, Landoni L, Casetti L, Tuveri M, Malleo G, Paiella S, Fontana M, Pea A, De Pastena M, Salvia R, Bassi C (2021). Pancreatic surgery during COVID-19 pandemic: major activity disruption of a third-level referral center during 2020. Updates Surg.

[4] COVIDSurg Collaborative (2021). Effect of COVID-19 pandemic lockdowns on planned cancer surgery for 15 tumour types in 61 countries: an international, prospective, cohort study. Lancet Oncol.

[5] Decreto Regionale Veneto Nr.120472 del 13.03.2020

[6] Nota Regione Veneto Nr. 474775 del 6/11/2020

[7] Marchegiani G, Paiella S, Malleo G, Landoni L, Tuveri M, Esposito A, Casetti L, De Pastena M, Fontana M, Salvia R, Bassi C (2020). Love (pancreatic surgery) in the time of cholera (COVID-19). Dig Surg.

[8] Hoyt D, Turner P. COVID-19: recommendations for management of elective surgical procedures. Available from: www.facs.org/covid-19/clinical-guidance.

[9] Persad G, Wertheimer A, Emanuel EJ (2009). Principles for allocation of scarce medical interventions. Lancet.

[10] Emanuel EJ, Persad G, Upshur R (2020). Fair allocation of scarce medical resources in the time of Covid-19. N Engl J Med.

[11] Presidenza del Consiglio dei Ministri - Segretariato Generale. Comitato Nazionale per la Bioetica. available at http://bioetica.governo.it/media/4079/pareri-cnb-su-covid-19-ita.pdf.

[12] SIAARTI SIMLA “Decisioni per le cure intensive in caso di sproporzione tra necessità assistenziali e risorse disponibili in corso di pandemia di COVID-19”. Available at https://www.siaarti.it/news/382977.

[13] Bassi C, Marchegiani G, Giuliani T, Di Gioia A, Andrianello S, Zingaretti CC, Brentegani G, De Pastena M, Fontana M, Pea A, Paiella S, Malleo G, Tuveri M, Landoni L, Esposito A, Casetti L, Butturini G, Falconi M, Salvia R (2020). Pancreatoduodenectomy at the Verona Pancreas Institute: the evolution of indications, surgical techniques and outcomes: a retrospective analysis of 3000 consecutive cases. Ann Surg.

[14] Marinelli V, Danzi OP, Mazzi MA, Secchettin E, Tuveri M, Bonamini D, Rimondini M, Salvia R, Bassi C, Del Piccolo L (2020). Prepare: preoperative anxiety reduction. One-year feasibility RCT on a brief psychological intervention for pancreatic cancer patients prior to major surgery. Front Psychol.

[15] Oxman D (2021). Allocation plans for crisis triage: how well would they actually work?. Cell Rep Med.

[16] Marchegiani G, Andrianello S, Nessi C, Sandini M, Maggino L, Malleo G, Paiella S, Polati E, Bassi C, Salvia R (2018). Neoadjuvant therapy versus upfront resection for pancreatic cancer: the actual spectrum and clinical burden of postoperative complications. Ann Surg Oncol.

[17] Yang YM, Tian XD, Zhuang Y (2005). Risk factor of pancreatic leakage after pancreaticoduodenectomy. World J Gastroenterol.

[18] Malleo G, Maggino L, Ferrone CR, Marchegiani G, Warshaw AL, Lillemoe KD, Bassi C, Castillo CF, Salvia R (2020). Reappraising the concept of conditional survival after pancreatectomy for ductal adenocarcinoma: a bi-institutional analysis. Ann Surg.

[19] Surci N, Ramera M, Borin A, Marchegiani G, Salvia R, Bassi C, Group SP (2020). Implementation of a strategic preoperative surgical meeting to improve the level of care at a high-volume pancreatic center: a before-after analysis of 1000 consecutive cases. Updates Surg.

[20] Kutikov A, Weinberg DS, Edelman MJ, Horwitz EM, Uzzo RG, Fisher RI (2020). A war on two fronts: cancer care in the time of COVID-19. Ann Intern Med.

[21] Hanna TP, King WD, Thibodeau S, Jalink M, Paulin GA, Harvey-Jones E, O'Sullivan DE, Booth CM, Sullivan R, Aggarwal A (2020). Mortality due to cancer treatment delay: systematic review and meta-analysis. BMJ.

[22] Oba A, Stoop TF, Lohr M, Hackert T, Zyromski N, Nealon WH, Unno M, Schulick RD, Al-Musawi MH, Wu W, Zhao Y, Satoi S, Wolfgang CL, Abu Hilal M, Besselink MG, Del Chiaro M, Pancreas Club EPCCPSAECoMIPSSGoPTfPCSGoPDAw, International Study Group on Cystic Tumors of the P (2020). Global survey on pancreatic surgery during the COVID-19 pandemic. Ann Surg.

[23] COVID-Surg Collaborative (2020). Delaying surgery for patients with a previous SARS-CoV-2 infection. Br J Surg.

[24] Glasbey JC, Nepogodiev D, Simoes JFF, Omar O, Li E, Venn ML, Pgdme K, Abou Chaar MK, Capizzi V, Chaudhry D, Desai A, Edwards JG, Evans JP, Fiore M, Videria JF, Ford SJ, Ganly I, Griffiths EA, Gujjuri RR, Kolias AG, Kaafarani HMA, Minaya-Bravo A, McKay SC, Mohan HM, Roberts KJ, San Miguel-Mendez C, Pockney P, Shaw R, Smart NJ, Stewart GD, Sundar Mrcog S, Vidya R, Bhangu AA, Collaborative CO (2021). Elective Cancer Surgery in COVID-19-Free Surgical Pathways During the SARS-CoV-2 Pandemic: an International, Multicenter, Comparative Cohort Study. J Clin Oncol.

[25] Carioli G, Malvezzi M, Bertuccio P, Boffetta P, Levi F, La Vecchia C, Negri E (2021). European cancer mortality predictions for the year 2021 with focus on pancreatic and female lung cancer. Ann Oncol.

[26] Hanna TP, King WD, Thibodeau S, Jalink M, Paulin GA, Harvey-Jones E, O'Sullivan DE, Booth CM, Sullivan R, Aggarwal A (2020). Mortality due to cancer treatment delay: systematic review and meta-analysis. BMJ.

[27] Corte di Cassazione, Sez. III, n. 5641 del 2018.

